# Gliflozins in Practice: Real-Life Use of Dapagliflozin and Empagliflozin in HFrEF Versus Clinical Trial Data

**DOI:** 10.3390/diagnostics16050769

**Published:** 2026-03-04

**Authors:** Massimo Mapelli, Rebecca Caputo, Massimo Valenti, Filippo Maria Rubbo, Elisabetta Salvioni, Irene Mattavelli, Arianna Galotta, Arianna Piotti, Fiorella Puttini, Laura Manfrin, Carlo Vignati, Simona Costantino, Piergiuseppe Agostoni

**Affiliations:** 1 Centro Cardiologico Monzino, IRCCS, 20138 Milan, Italy; rebecca.caputo@ccfm.it (R.C.); elisabetta.salvioni@ccfm.it (E.S.); irene.mattavelli@ccfm.it (I.M.); arianna.galotta@ccfm.it (A.G.); arianna.piotti@ccfm.it (A.P.); fioreputtini@gmail.com (F.P.); carlo.vignati@ccfm.it (C.V.); simona.costantino@unimi.it (S.C.); piergiuseppe.agostoni@ccfm.it (P.A.); 2 Department of Clinical Sciences and Community Health, Cardiovascular Section, University of Milan, 20122 Milan, Italy; 3 ERN GUARD-Heart Center for Diagnosis and Treatment of Cardiomyopathies, Cardiovascular Department, ASUGI, University of Trieste, 34100 Trieste, Italyfilippomariarubbo@gmail.com (F.M.R.); manfrinlaura3@gmail.com (L.M.)

**Keywords:** heart failure, SGLT2i, dapagliflozin, empagliflozin, real-life

## Abstract

**Background:** Sodium/glucose cotransporter-2 inhibitors (SGLT2is), such as dapagliflozin and empagliflozin, are currently a standard therapy for heart failure (HF) patients. We report the real-world use of SGLT2is in a monocentric cohort of HF patients with reduced ejection fraction (HFrEF) and improved ejection fraction (HFimpEF), comparing patient characteristics and outcomes with those observed in large-scale randomized clinical trials (RCTs). **Methods:** We retrospectively analyzed a cohort of 370 stable patients with HFrEF or HFimpEF who initiated therapy with dapagliflozin or empagliflozin between June 2019 and November 2023. Baseline data, including medical history, concomitant diseases, therapy, laboratory tests, echocardiographic results and cardiopulmonary exercise tests (CPETs), were collected at the start of the therapy with SGLT2is. After a median period of 18 months, follow-up data on treatment adherence, adverse events, hospitalizations, and mortality were also reviewed. A comparison was made between patients taking dapagliflozin and those taking empagliflozin and then individual populations were compared with those from the trials. **Results:** Among 370 patients (81% HFrEF, 19% HFimpEF), 276 received dapagliflozin and 94 empagliflozin. Empagliflozin patients were older, had higher NYHA class and LVEF, and higher incidence of diabetes, while dapagliflozin users had greater use of sacubitril/valsartan and mineralocorticoid receptor antagonists. Both groups were older than the RCT cohorts. Dapagliflozin patients had LVEF comparable to DAPA-HF, while empagliflozin patients had higher LVEF than EMPEROR-Reduced. HF hospitalizations were more frequent in the real-world groups, but mortality was lower than in RCTs. The composite outcome of death and worsening HF was higher in the real-world dapagliflozin cohort vs. DAPA-HF but similar between the real-world empagliflozin cohort and EMPEROR-Reduced. **Conclusions:** In this real-world cohort, the use of empagliflozin was associated with cardio-nephro-metabolic comorbidities and dapagliflozin being prescribed more frequently for patients with isolated cardiac symptoms. While outcomes were generally favorable, they differed from those seen in RCTs, highlighting the importance of real-world data in understanding the practical application of these therapies.

## 1. Introduction

After the introduction of sacubitril/valsartan, sodium/glucose cotransporter-2 inhibitors (SGLT2is, gliflozins) were the first medications evaluated in large-scale trials to assess their efficacy in the treatment of heart failure (HF) with reduced ejection fraction (HFrEF).

Beyond their well-established efficacy in treating diabetes, SGLT2is have demonstrated cardiovascular (CV) benefits through mechanisms that remain not fully understood and, in any case, independent of their glycosuric effect. The primary mechanisms underlying these effects include enhanced endothelial function, protection of the endothelium from intracellular reactive oxygen species production, increased nitric oxide bioavailability, reduced microvascular dysfunction, reduced insulin resistance, and both visceral and subcutaneous adipose tissue [1]. Interestingly, these pathophysiological mechanisms translate into tangible effects on parameters commonly used in clinical practice to assess the effectiveness of a pharmacological therapy, such as left ventricular reverse remodeling or functional capacity parameters [2].

The DAPA-HF [3] and EMPEROR-Reduced [4] trials demonstrated the efficacy of SGLT2is in reducing cardiovascular death and hospitalization for HF in patients with HFrEF. Subsequently, the DELIVER [5] and EMPEROR-Preserved [6] trials demonstrated a reduction in hospitalization for HF as well as among patients with heart failure with preserved ejection fraction (HFpEF). Based on this evidence, in 2021 SGLT2is were recommended by European guidelines for HFrEF and were subsequently extended in 2023 to all patients with a HF diagnosis, regardless of Left Ventricular Ejection Fraction (LVEF) [7,8]. SGLT2is have since become the fourth pillar in the treatment of HFrEF, so that their use has increased markedly in recent years [1].

The aim of our study was to describe the use of SGLT2is (i.e., dapagliflozin and empagliflozin) in a monocentric, real-world cohort of patients with current or previously diagnosed HFrEF and to explore potential differences compared to the respective clinical trials.

## 2. Materials and Methods

We retrospectively analyzed a cohort of consecutive stable HFrEF and heart failure with improved ejection failure (HFimpEF) patients who started therapy with an SGLT2i, dapagliflozin or empagliflozin, in accordance with the European Society of Cardiology guidelines for HF [8], between June 2019 and November 2023 at the Heart Failure Unit of the Centro Cardiologico Monzino. In order to provide a reliable representation of real-world clinical practice, no restrictive inclusion or exclusion criteria were applied, therefore reflecting the inherent clinical heterogeneity of the patient population.

Baseline data, obtained from medical records, were collected from the date of prescription of SGLT2is and included demographic data, medical history, concomitant diseases, ongoing therapies with other treatment and clinical characteristics. These data also incorporate the results from blood tests, echocardiography and cardiopulmonary exercise testing (CPETs) performed at our site, when available within 3 months. We also collected data regarding follow-up duration (defined as last contact with the site), creatinine values, treatment discontinuation, any adverse events, and any cases of hospitalization due to heart failure or death, through medical records or by telephone contact. The Metabolic Exercise Cardiac Kidney Indexes (MECKI) score, which included 6 relevant prognostic parameters—hemoglobin (Hb), LVEF, renal function assessed by MDRD equation, serum sodium (Na+), peak oxygen intake (PeakVO2), and Ventilation to Carbon Dioxide production (VE/VCO2) slope—was calculated as previously described in patients with all the variables available [9].

The present research protocol complies with the World Medical Association’s Declaration of Helsinki and was approved by the Centro Cardiologico Monzino Ethical Committee (R 11637-22 CCM 1756; approval date: 6 April 2022). Each subject provided written informed consent to participate in this study. Study data were collected and managed using REDCap electronic data capture tools hosted at Centro Cardiologico Monzino IRCCS [10,11]. REDCap (Research Electronic Data Capture) is a secure, web-based software platform.

### Statistical Analyses

Since this was an observational study, the sample size was determined by the patients available in our clinical practice during the study period. However, post hoc power considerations confirmed that our sample of 370 patients (276 dapagliflozin, 94 empagliflozin) provides 80% statistical power to detect a between-group difference of 0.335 SD (alpha = 0.05). Regarding the comparison with trials, no sample size calculation was feasible because we lacked individual patient-level data.

Continuous variables were described as the mean ± standard deviation (SD) in cases of normal distribution, and as the median and interquartile range (IQR) in cases of non-normal distribution. Categorical variables were expressed as numbers and percentages. For continuous variables, differences between baseline and follow-up were assessed with paired t-tests or non-parametric tests (Wilcoxon Signed-Rank test), as appropriate. Differences between subgroups of patients were tested using unpaired t-tests, Mann–Whitney U tests, Chi-square or Fisher’s exact tests, depending on the data type and distribution. To compare the characteristics of patients included in this study with those enrolled in the DAPA-HF and EMPEROR-Reduced trials, independent samples t-tests or non-parametric tests, Chi-square tests or Fisher’s exact tests were used, as appropriate. Kaplan–Meier (KM) curves from the included trials were digitized using PlotDigitizer software (available online at https://plotdigitizer.com). Individual patient-level survival data were reconstructed using the “KM_reconstruct” function from the reconstructKM package in R (version 4.3.1); this function allows for the reconstruction of digitized KM curves into individual patient data. The reconstructed data were subsequently used to estimate survival curves and calculate hazard ratios (HRs) using Cox proportional hazards models.

## 3. Results

This study included 370 consecutive patients with stable HFrEF (*n* = 224, 81%) and HFimpEF (*n* = 52, 19%) who initiated treatment with dapagliflozin (*n* = 276) or empagliflozin (*n* = 94) at our HF Unit. Their data were compared with DAPA-HF (*n* = 2373) and EMPEROR-Reduced (*n* = 1863) trials, respectively. Patients were followed for a median period of 19 months (15–23), with 292 subjects completing follow-up through a site visit, 48 by phone call, and the remaining 30 were considered lost to follow-up.

### 3.1. Comparison of Real-World Cohorts

First, we compared the two real-life populations with each other (Table 1). At baseline, the real-world empagliflozin patients were older (73 ± 11 vs. 70 ± 13 years, *p* = 0.014) and had a different NYHA class distribution with more patient in NYHA class III (31% vs. 17.6%, *p* = 0.033) compared to the dapagliflozin group. Additionally, the empagliflozin cohort exhibited a higher LVEF (34 ± 8% vs. 32 ± 8%, *p* = 0.015) and lower LV volumes (End-Diastolic Volume (EDV) 157 [118–202] vs. 183 [145–234] mL, *p* = 0.003; End-Systolic Volume (ESV) 105 [73–133] vs. 122 [90–168] mL, *p* = 0.002). Estimated pulmonary pressure (PAPs) was higher in patients treated with empagliflozin (38 [29–47] vs. 32 [26–40] mmHg, *p* = 0.003). In both populations there was a small proportion of patients with HFimpEF at baseline (31 (11%) in dapagliflozin, 15 (16%) in empagliflozin *p* = 0.1460).

**Figure 1:**
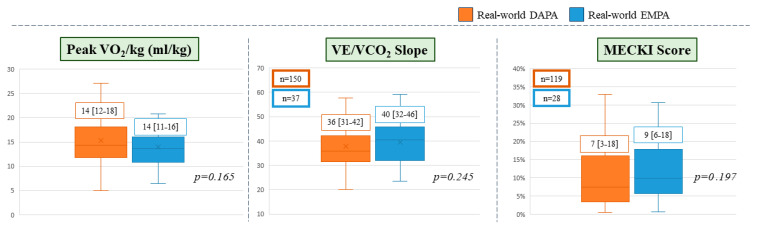
CPET and MECKI score data in real-world populations. Box plots illustrate the baseline distribution of three parameters in patients treated with dapagliflozin (DAPA, orange) and empagliflozin (EMPA, blue). Left panel presents median values of Peak VO_2_ (mL/kg), the middle panel shows median values of VE/VCO_2_ slope and the right panel illustrates median values of MECKI score (%). Abbreviations: CPETs: cardiopulmonary exercise tests, MECKI: Metabolic Exercise Cardiac Kidney Indexes, VO_2_/kg; VE/VCO_2_: Ventilation to Carbon Dioxide; DAPA: dapagliflozin; EMPA: empagliflozin.

Differences were also observed in pre-treatment laboratory parameters, with the empagliflozin group showing lower hematocrit and Hb levels and higher creatinine values compared to the dapagliflozin group, while no significant difference in NT-proBNP levels was observed (Table 1). In terms of therapy, the real-life dapagliflozin group exhibited a higher prevalence of concomitant treatment with sacubitril/valsartan (82% vs. 66%, *p* = 0.001) and mineralocorticoid receptor antagonists (MRAs) (79% vs. 69%, *p* = 0.047). The empagliflozin group also exhibited a higher proportion of patients with diabetes. Regarding loop diuretics therapy, 28.4% of patients were not receiving treatment (29.6% with dapagliflozin vs. 22% with empagliflozin). In the overall study population, 86.8% of all patients were receiving a dose of 50 mg or less. Specifically, the most frequent daily dosages were 25 mg (29.0% for dapagliflozin and 27.7% for empagliflozin) and 50 mg (19.2% for dapagliflozin and 17.0% for empagliflozin). Finally, in the subgroups of patients who completed the baseline evaluation with a maximal CPET (*n* = 187 (45%), 150 (80%) in the dapagliflozin group and 37 (20%) in the empagliflozin group), no significant differences regarding exercise capacity were observed with similar PeakVO_2_ (Figure 1) and VE/VCO_2_ slope values (dapagliflozin 36 [31–42] vs. empagliflozin 40 [32–46], *p* = 0.245). In the dapagliflozin group (*n* = 119), the 2-year estimated cardiovascular risk (death or urgent heart transplant or left ventricle assist device implantation) by MECKI score was 7% [3–18] in the dapagliflozin cohort and 9% [6–18] in the empagliflozin group (*p* = 0.197).

### 3.2. Real-World Cohorts Compared to Trials

A further comparison was made between our cohorts and the respective RCT baseline characteristics (Table 2), considering comparable median follow-up duration between real-world (19 months) and trial populations (DAPA-HF 18.2 months and EMPEROR-Reduced 16 months). In both cases our real-world populations were significantly older, while no differences were observed in gender distribution. The dapagliflozin-treated population had a similar LVEF to the DAPA-HF trial (32 ± 8% vs. 31 ± 7% in DAPA-HF, *p* = 0.070), while the empagliflozin-treated population exhibited a significantly higher LVEF compared to the EMPEROR-Reduced trial (34 ± 8% vs. 28 ± 6% in EMPEROR-Reduced *p* = 0.0001).

**Figure 2:**
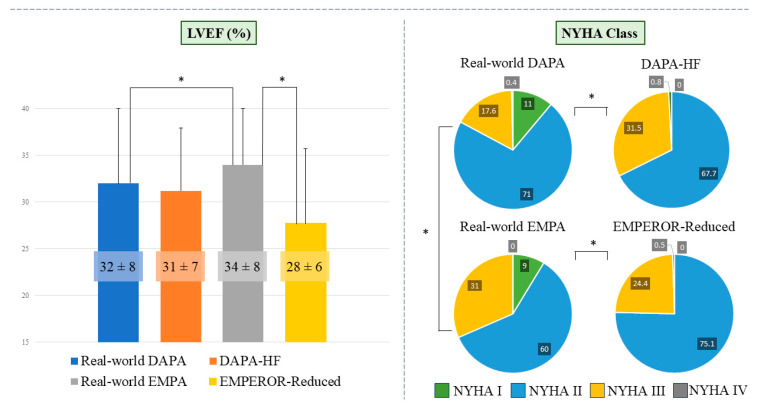
LVEF and NYHA class data in real-world and RCT populations. The bar plot shows mean ejection fraction (%) values in patients treated with dapagliflozin (blue) and empagliflozin (gray), comparing real-world cohorts with those enrolled in the DAPA-HF (orange) and EMPEROR-Reduced (yellow) trials. Pie charts illustrate the distribution of NYHA functional classes across the same groups. Abbreviations: LVEF: Left Ventricular Ejection Fraction; NYHA: New York Heart Association; RCT: randomized clinical trials; DAPA: dapagliflozin; EMPA: empagliflozin; HF: heart failure. Statistically significant *p*-values are indicated with *.

In terms of NYHA functional classes, as shown in Table 2 and Figure 2, our dapagliflozin population showed a higher percentage of patients in classes I and II than DAPA-HF, indicating better clinical status. Similarly, the empagliflozin population showed a higher percentage of NYHA class I patients, but otherwise there is a lower percentage in classes II and III than EMPEROR-Reduced.

**Figure 3:**
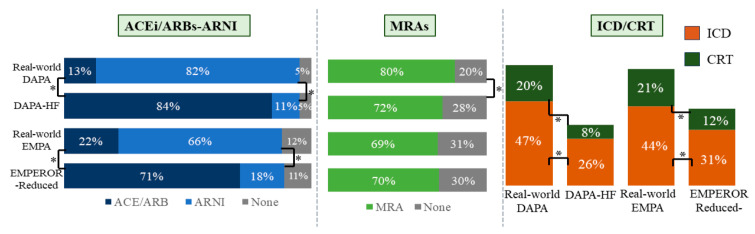
HF therapy and devices data in real-world and RCT populations. The left panel presents a comparison of pharmacological therapy: percentages of patients on ACEi/ARBs (blue) and ARNI (light blue). The central panel illustrates percentages of patients on MRAs (light green). The right panel analyzes the device use (ICD and CRT), showing the percentages of patients with ICD (orange) and CRT (green). Abbreviations: HF: heart failure; RCT: randomized clinical trials; DAPA: dapagliflozin; EMPA: empagliflozin; ACEi: Angiotensin-Converting Enzyme Inhibitor; ARB: Angiotensin Receptor Blocker; MRA: Mineralocorticoid Receptor Antagonist; ICD: Implantable Cardioverter-Defibrillator; CRT: Cardiac Resynchronization Therapy. Statistically significant *p*-values are indicated with *.

Regarding drug therapy, beta-blockers prescription rates were comparable between the dapagliflozin and empagliflozin groups and their respective trials, but the population prescribed with dapagliflozin was more bradycardic than in the DAPA-HF population. Significant differences were observed in the prescription of sacubitril/valsartan, which is higher in both real-world populations than in the trials (Figure 3). Accordingly, Angiotensin-Converting Enzyme Inhibitors and Angiotensin Receptor Blockers are more prescribed in the trial cohorts. Additionally, in the DAPA HF population more patients were treated with diuretics (DAPA-HF 93.4% vs. 70.4% in the real-life group, *p* < 0.0001) and fewer with MRAs (DAPA-HF 71% vs. 79% in the real-life group, *p* = 0.0068).

The prevalence of diabetic patients differed between the real-life population and the DAPA-HF cohort, but it was similar among patients treated with empagliflozin compared to the EMPEROR-Reduced trial. In addition, the real-life patients showed a higher rate in previous device implantation (i.e., Implantable Cardioverter-Defibrillator (ICD) and Cardiac Resynchronization Therapy (CRT) compared with the trials for both the drugs) (Figure 3). Both study populations exhibited significantly lower levels of NTproBNP compared to their respective trial (Table 2).

We also analyzed follow-up data available after a median period of 19 months (15–23) of treatment with dapagliflozin and empagliflozin to be compared with RCT. Regarding the change in creatinine from baseline, there were no differences between the real-life and trial populations. Fewer discontinuations were observed in the dapagliflozin group than in the RCT group (6% vs. DAPA HF: 11%, *p* < 0.0183).

**Figure 4:**
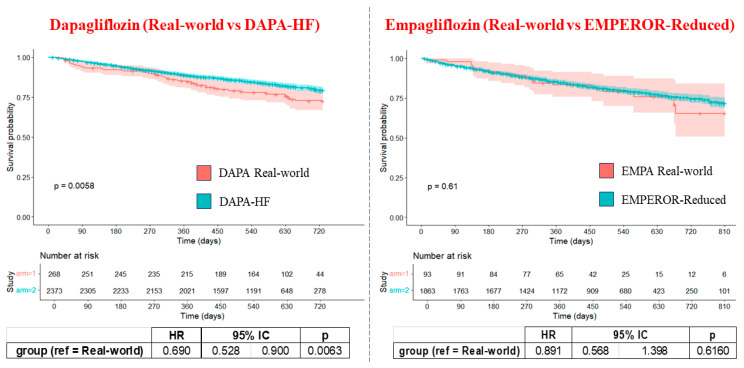
Kaplan–Meier curves in the real-life versus RCT populations. Kaplan–Meier curves showing event-free survival over time between real-world populations in red and RCT populations in blue. Abbreviations: RCTs: randomized clinical trials; DAPA: dapagliflozin; EMPA: empagliflozin, HF, heart failure.

According to clinical outcome, follow-up data showed that both trials reported fewer HF hospitalizations (dapagliflozin: 50 (19.6%) vs. DAPA-HF: 237 (11.5%), *p* < 0.0001; empagliflozin: 16 (19%) vs. EMPEROR-Reduced: 246 (13%), *p* < 0.0001) and more deaths compared to the real-world population (dapagliflozin: 22 (8%) vs. DAPA HF 503 (21%) *p* < 0.0001; empagliflozin: 8 (9%) vs. EMPEROR-Reduced:436 (23%) *p* = 0.0013). Finally, we also evaluated the composite outcome of death and worsening HF events, including hospitalization, urgent visits, or administration of diuretics or intravenous inotropes. This outcome shows no difference between the two real-world populations, but it is higher in the dapagliflozin group compared to DAPA-HF (67 (24%) vs. 386 (16%), *p* = 0.0004) and not different in real-world empagliflozin compared to EMPEROR-Reduced (21 (22%) vs. 361 (19%) *p* = 0.479), as shown in Table 3. Kaplan–Meier curves comparing real-world and trial populations show that patients in the DAPA-HF trial had a significantly lower risk of events compared to our real-world cohort (HR 0.69, 95% CI 0.53–0.90, *p* = 0.0063). No significant difference was observed for empagliflozin compared to the EMPEROR-Reduced trial (HR 0.89, 95% CI 0.57–1.40, *p* = 0.62) (Figure 4).

## 4. Discussion

This study provided insights into the differences in prescription and population characteristics of two prescribed SGLT2is, dapagliflozin and empagliflozin, in an HFrEF and HFimpEF real-world cohort of patients on optimal medical therapy enrolled at our HF Unit, Centro Cardiologico Monzino.

While other datasets exist, our study adds value by providing a detailed monocentric characterization that includes parameters often missing in larger registries, such as CPET data and the MECKI score. Furthermore, our analysis uniquely compares real-world data with reconstructed individual patient-level data from the DAPA-HF and EMPEROR-Reduced trials, allowing for a more granular comparison of outcomes like HF hospitalizations and mortality in a specific Italian clinical context.

In our center dapagliflozin was prescribed more than empagliflozin, despite what would have been expected from the shared group class mechanism of action [12] of the two molecules and the ongoing literature on the prevalence of the two drugs in this population [13,14,15,16]. This difference might be explained by the fact that dapagliflozin was the first authorized SGLT2i prescription drug for symptomatic chronic HF patients in Italy, with empagliflozin shortly following. Other factors might explain this difference in prevalence of the two drugs within our cohort, such as preference of the prescribing physician and pharmaceutical marketing within the city and the center [17,18], but no data could be gathered on the prevalence of drug prescription among different physicians.

Empagliflozin was more frequently prescribed than dapagliflozin in patients with a previous diagnosis of diabetes at baseline. There is a significantly lower percentage of diabetic patients in the real-world dapagliflozin population compared to its trial population (*p*-value < 0.0001). As this appears as an unexpected result at first, some reasons why our real-world dapagliflozin population had a significantly lower percentage of patients with a diagnosis of diabetes compared to the DAPA-HF trial and established estimates on the prevalence of diabetes within HF patients might be hypothesized. First, the real-world dapagliflozin group has a significantly different distribution of HF etiologies (*p*-value < 0.0001) with a lower percentage of ischemic patients compared to the trial population, thus partially explaining the lower prevalence of patients with diabetes observed [19]. Second, the percentage of patients with diabetes within the combined two real-world groups is 19%, a value similar to that observed in previous years in our center HF population [20]. This suggests a difference in prescription preference among real-world HF patients with diabetes at our center, favoring empagliflozin over dapagliflozin, rather than a deviation from expected epidemiology or published trials.

Empagliflozin being more frequently prescribed in diabetic patients compared to dapagliflozin in our real-world population might be explained by the different outcomes of the EMPA-REG OUTCOME and DECLARE-TIMI 58 trials [21,22], which showed a reduction in MACE and a reduction only in hospitalizations respectively, even though these results may be due to differences in cohort populations [21,22,23].

Notably, in our real-world population empagliflozin is also prescribed more frequently in higher NYHA class patients, with higher values of PASP, creatinine, and concomitant use of antidiabetic medications, with lower values of hematocrit, Hb and percentages of patients taking MRAs and sacubitril/valsartan, depicting a patient more symptomatic and comorbid than the counterpart being prescribed dapagliflozin. Although there is no significant difference in eGFR between the two groups, some differences in kidney function might be suspected given the higher creatinine value, the lower hemoglobin value, and the lower percentage of patients taking MRAs in the real-world empagliflozin population. A preference for empagliflozin over dapagliflozin in patients with kidney dysfunction might then be explained with the different prescription regulation for the two drugs, with empagliflozin having been approved for lower eGFR than dapagliflozin (25 mL/min vs. 30 mL/min) because of respective trial designs. On the other hand, in our cohort dapagliflozin is prescribed in patients with more negatively remodeled left ventricular chambers, given the larger volumes and the lower LVEF. Taken together these data suggest that in our population dapagliflozin was prescribed in patients with an “isolated” (or at least predominant) cardiac presentation, while empagliflozin was prescribed in more complex and comorbid “cardio-nephro-metabolic” patients, despite no known evidence of the advantages of one drug or the other in these groups.

After a median follow-up of 19 months, outcomes are similar between the two real-world groups but differ from those of the respective trials for a lower incidence of death despite a higher incidence of HF hospitalizations and HF hospitalization-driven composite outcome in the real-world dapagliflozin cohort compared with the DAPA-HF population. A similar finding was observed in the real-world empagliflozin cohort, despite not reaching a statistically significant difference, probably due to the lower number of patients. A lower implementation of optimal medical therapy (as suggested by a higher prescription of sacubitril/valsartan, MRAs and beta-blockers and by the higher presence of CRT/ICD devices for both real-world groups compared to the two trials and to most HF registries [15,24,25]), and a more advanced stage of disease as highlighted by higher values of NT-proBNP in the trial populations compared to our real-world groups are probably likely associated with the differences in mortality despite the population of our study being older. Notably, the marked difference in ARNI use compared to the pivotal trials is mainly due to our more recent recruitment, which coincided with the full commercialization of the drug and its class I recommendation in the 2021 ESC guidelines. Higher incidence of hospitalization in HFrEF patients compared to those observed in the DAPA-HF trial is vastly reported in the literature, with values like those of our real-world group. Also, the reduction in mortality observed alongside better therapy implementation might account for the higher prevalence of HF hospitalizations among our real-world cohorts, as optimized therapy extends survival in elderly and fragile patients, increasing their exposure time to non-fatal events related to their clinical complexity.

Finally, the CPET results are coherent with the observed outcomes in the two real-world groups as no differences between the respective populations emerged even if a 2% higher MECKI score value was observed in the empagliflozin cohort.

Our study was subject to several key limitations. This was a single-center observational retrospective study with a small sample size. Importantly, our real-world cohort was not matched with trial populations; the inherent differences in study design and patient characteristics between real-world evidence and pivotal trials mean these groups are not directly comparable. As such, the findings should be interpreted as clinical associations reflecting real-world management rather than evidence of direct causality due to the different nature of both studies. At the beginning of this study, only one of the two drugs was available in our country for HF patients. In our study HFimpEF patients were included and comprised a relevant part of our population; nonetheless SGLT2is proved their efficacy on a broad spectrum of HF ejection fractions [5,6], making our results more comprehensive and up to date. Adherence could not be evaluated, as pharmacy dispensing data are not available in this real-world setting.

Moreover, the absence of additional patient-level clinical information limits the ability of the reconstructed dataset to account for potentially relevant clinical factors that may have influenced the observed outcomes. Propensity score matching was not applied in this study. However, the primary objective was to explore the “real-life” application of SGLT2is and identify how the clinical profile of patients treated in daily practice differs from those selected for pivotal trials. We believe that by presenting the unmatched data, we provide a more transparent view of the actual clinical landscape and the challenges of translating trial results into practice. Finally, only a small proportion of our population performed a CPET, possibly underpowering the observed results.

## 5. Conclusions

This real-world study highlights notable differences in the clinical profiles of patients prescribed dapagliflozin versus empagliflozin, suggesting that the choice of SGLT2i may reflect distinct patient characteristics rather than being interchangeable. Dapagliflozin was more commonly prescribed in patients with an isolated cardiac phenotype, while empagliflozin was more frequently used in patients with diabetes and a higher burden of cardiovascular–renal–metabolic comorbidities.

Despite generally favorable outcomes, event rates for hospitalizations and mortality differed from those observed in RCT, reflecting clinical practice rather than a direct comparison. These observational findings underscore the importance of real-world data to complement trial evidence and guide clinical decision-making in everyday practice.

## Figures and Tables

**Table 1:** Characteristics of real-life cohorts.

		Real-Life DAPA		Real-Life EMPA		*p*-Value
		*n*	media ± SD, *n* (%)	*n*	media ± SD, *n* (%)	
Age (years)		276	70 ± 12.6	94	73 ± 11	0.014
Sex		276		94		
	Male		200 (72.4%)		70 (74%)	0.705
	Female		76 (27.6%)		24 (26%)	
BMI (kg/m^2^)		250	26.3 ± 4.2	82	27 ± 4	0.588
NYHA		262		92		0.033
	I		28 (11%)		8 (9%)	
	II		187 (71%)		55 (60%)	
	III		46 (17.6%)		29 (31%)	
	IV		1 (0.4%)			
Etiology		276		94		0.2824
	Ischemic		133 (48%)		52 (55%)	
	Non Ischemic		143 (52%)		42 (44%)	
HFimpEF (*n*)			31 (11%)		21 (16%)	0.1460
LVEF (%)		267	32 ± 8	85	34 ± 8	0.015
EDV (mL)		225	183 (145–234)	69	157 (118–202)	0.003
ESV (mL)		225	122 (90–168)	68	105 (73–133)	0.002
PASP (mmHg)		210	32 (26–40)	66	38 (29–47)	0.003
SBP (mmHg)		253	120 ± 17	76	121 ± 17	0.93
Heart rate (bpm)		217	69 ± 14	74	69 ± 15	0.92
Hemoglobin (g/dL)		243	13.7 (13–15)	87	12.6 (11.1–15.5)	0.004
Hematocrit (%)		180	41 (37–44)	62	38 (33–42)	0.005
Creatinine (mg/dL)		256	1.25 ± 0.7	89	1.4 ± 0.6	0.043
EGFR (mL/min)		256	62 ± 22	89	59 ± 61	0.65
NT-proBNP (pg/mL)		157	1047 (482–2559)	41	1393 (507–3216)	0.415
Diabetes		273	42 (15%)	94	27 (29%)	<0.001
Hba1c (%)		93	6.6 ± 4.7	21	11 ± 14	0.207
PM		273	43 (16%)	94	11 (12%)	0.339
ICD		274	130 (47%)	94	41 (44%)	0.521
CRT		275	54 (20%)	94	20 (21%)	0.732
ACE		273	20 (7%)	93	10 (11%)	0.298
ARB		272	16 (6%)	93	10 (11%)	0.115
Sacubitril/Valsartan		274	224 (82%)	93	61 (66%)	0.001
Beta-blocker		275	264 (96%)	94	89 (95%)	0.588
Loop diuretics		274	193 (70.4%)	94	73 (78%)	0.177
Statin		275	165 (60%)	94	65 (69%)	0.114
MRA		274	217 (79%)	94	65 (69%)	0.047
Antiplatelet		273	126 (46%)	94	54 (57%)	0.059
Anticoagulation therapy		274	120 (44%)	94	37 (39%)	0.453
Digitalis		273	21 (8%)	94	5 (5%)	0.439
Amiodarone		274	105 (38%)	94	32 (34%)	0.459
Oral antidiabetic		275	21 (8%)	94	16 (17%)	0.009
Insulin		275	9 (3%)	94	13 (14%)	<0.001
Creatinine at follow-up (mg/dL)		190	1.3 ± 0.5	61	1.5 ± 0.7	0.015
EGFR at follow-up (mL/min)		184	55 ± 21	59	50 ±24	0.1259
Composite outcome		276	67 (24%)	94	21 (22%)	0.7039

Abbreviations: BMI: Body Mass Index; NYHA: New York Heart Association; HFimpEF: heart failure with improved ejection fraction; LVEF: Left Ventricular Ejection Fraction; HbA1c: hemoglobin A1c; EDV: End-Diastolic Volume; ESV: End-Systolic Volume; PASP: pulmonary artery systolic pressure; SBP: Systolic Blood Pressure; EGFR: Estimated Glomerular Filtration Rate; NT-proBNP: N-terminal pro b-type natriuretic peptide; PM: Pacemaker; ICD: Implantable Cardioverter-Defibrillator; CRT: Cardiac Resynchronization Therapy; ACEi: Angiotensin-Converting Enzyme Inhibitor; ARB: Angiotensin Receptor Blocker; MRA: mineralocorticoid receptor antagonist. Statistically significant *p*-values are shown in yellow.

**Table 2:** Comparison between real-life populations and randomized trials.

		Real-Life DAPA	DAPA HF	*p*-Value	Real-Life EMPA	EMPEROR	*p*-Value
		*n* = 276	*n* = 2373		*n* = 94	*n* = 1863	
Age(years)		70 ± 13	66 ± 11	<0.0001	73 ± 11	67 ± 11	<0.0001
Sex	Male	200 (72.4%)	1809 (76%)	0.1662	70 (74%)	1426(76.5%)	0.6437
	Female	76(27.6%)	564 (23.8%)		24 (26%)	437 (23.5%)	
BMI(kg/m^2^)		26 ± 4	28 ± 6	<0.0001	27 ± 4	28 ± 6	0.104
NYHA	I	28 (11%)	0	<0.0001	8 (9%)	0	<0.0001
	II	187 (71%)	1606 (67.7%)		55 (60%)	1399 (75.1%)	
	III	46 (17.6%)	747 (31.5%)		29 (31%)	455 (24.4%)	
	IV	1 (0.4%)	20 (0.8%)			9 (0.5%)	
Etiology	Ischemic	133 (48%)	1316 (55.5%)	0.0252	52 (55%)	983 (52.8)	0.6724
	Non Ischemic	143 (52%)	1057 (46%)		42 (44%)	880 (47.2)	
LVEF(%)		32 ± 8	31.2 ± 6.7	0.070	34 ± 8	28 ± 6.0	<0.0001
SBP (mmHg)		120 ± 17	122.0 ± 16.3	0.064	121 ± 17	122.6 ± 15.9	0.391
Heart rate(bpm)		69 ± 14	71.5 ± 11.6	0.003	69 ± 15	71.0 ± 11.7	0.154
EGFR (mL/min)		62 ± 22	66.0 ± 19.6	0.0053	59 ± 61	61.8 ± 21.7	0.129
NT-proBNP (pg/mL)		1047 (482-2559)	1428 (857–2655)	<0.0001	1393 (507-3216)	1887 (1077–3429)	0.0062
ICD		130 (47%)	622 (26%)	<0.0001	41 (44%)	578 (31%)	0.0104
CRT		54 (20%)	190 (8%)	<0.0001	20 (21%)	220 (12%)	0.0063
ACE		20 (7%)	1332 (56.1%)	<0.0001	10 (11%)		
ARB		16 (6%)	675 (28%)	<0.0001	10 (11%)	1314 (71%)	<0.0001
Sacubitril/Valsartan		224 (82%)	250 (10.5%)	<0.0001	61 (66%)	340 (18%)	<0.0001
Bblocker		264 (96%)	2278 (96%)	0.9978	89 (95%)	1765 (95%)	1
Diuretics		193 (70.4 %)	2216 (93.4%)	<0.0001	73 (78%)		
MRA		217 (79%)	1696 (71.5%)	0.0068	65 (69%)	1306 (70%)	0.844
Insulin		9 (3%)	274/993 (27.6%)	<0.0001	13 (14%)	664 (35.6)	<0.0001
Diabetes		42 (15%)	993 (41.8%)	<0.0001	27 (29%)	577 (31.0)	0.6452
History of HF hospitalisation		165 (60%)	1124 (47.4%)	<0.0001	84 (89%)	664 (35.6)	<0.0001
Discontinuation		15 (6%)	249 (11%)	0.0183	9 (11%)		
Adverse event	Renal adverse event	3 (21%)	153 (97.5%)	<0.0001	1 (10%)		
	Major hypoglycemia	1 (7%)	4 (2.5%)				
	Other	10 (71%)	0		8 (90%)		

Abbreviations: BMI: Body Mass Index; NYHA: New York Heart Association; LVEF: Left Ventricular Ejection Fraction; PSB: Systolic Blood Pressure; EGFR: Estimated Glomerular Filtration Rate; NT-proBNP: N-terminal pro b-type natriuretic peptide; ICD: Implantable Cardioverter-Defibrillator; CRT: Cardiac Resynchronization Therapy; ACEi: Angiotensin-Converting Enzyme Inhibitor; ARB: Angiotensin Receptor Blocker; MRA: Mineralocorticoid Receptor Antagonist; HF: heart failure. Statistically significant *p*-values are shown in light red.

**Table 3:** Comparison of outcomes between our cohorts and randomized trials.

		Real-Life DAPA	DAPA HF	*p*-Value	Real-Life EMPA	EMPEROR	*p*-Value
		*n* = 276	*n* = 2373		*n* = 94	*n* = 1863	
HF hospitalization		50 (19.6%)	237 (11.5%)	<0.0001	16 (19%)	246 (13.2)	0.1376
Death		22 (8%)	503 (21%)	<0.0001	8 (9%)	436 (23%)	0.00013
Death cause				0.6969			1
	CV death	9 (41%)	227 (45%)		3 (38%)	187 (43%)	
	Other	13 (59%)	276 (55%)		5 (62%)	249 (57%)	
Composite outcome		67 (24%)	386 (16%)	0.0004	21 (22%)	361 (19%)	0.479

Abbreviations: HF: heart failure; CV: cardiovascular. Statistically significant *p*-values are shown in light red.

## Data Availability

Repository of raw data available after acceptance: www.zenodo.org.
